# Impact of coronavirus disease (COVID-19) pandemic on physical activity of patients with cardiac implantable electronic devices—A remote monitoring study

**DOI:** 10.1371/journal.pone.0269816

**Published:** 2022-08-12

**Authors:** Moritz T. Huttelmaier, Alexander Gabel, Maria Seewald, Carsten Jungbauer, Stefan Frantz, Stefan Störk, Thomas H. Fischer

**Affiliations:** 1 Medizinische Klinik und Poliklinik I, Universitätsklinikum Würzburg, Würzburg, Germany; 2 Institut für Informatik, Martin-Luther-Universität Halle-Wittenberg, Halle, Germany; 3 Klinik und Poliklinik für Innere Medizin II, Universitätsklinikum Regensburg, Regensburg, Germany; 4 Deutsches Zentrum für Herzinsuffizienz (DZHI), Universität und Universitätsklinikum Würzburg, Würzburg, Germany; Universitatsklinikum Wurzburg, GERMANY

## Abstract

**Objectives:**

The study aims to investigate the impact of COVID-19 pandemic on physical activity and frequency of implantable cardioverter-defibrillator (ICD) therapies of patients with cardiac implantable electronic devices.

**Methods and results:**

Physical activity, heart rate and ICD-therapies were assessed via routine remote monitoring over two years. We focussed on a 338-day period during COVID-19 pandemic that was divided in 6 time-intervals defined by public health interventions and compared to the previous regular year. Paired nonparametric longitudinal analysis was performed to detect differences between time-intervals. To model effects of age, sex and time we applied a nonparametric ANOVA-type-statistic. 147 patients with cardiac implantable electronic devices were analysed. Longitudinal analysis of physical activity in 2019 and 2020 showed a specific weekly and seasonal pattern. Physical activity was reduced during the pandemic (mean daily physical activity 2019: 12.4% vs. 2020: 11.5%; p<0.0001) with the strongest reductions (fold changes 0.885/0.889, p<0.0001/p<0.0001) during the two lockdown-periods. In older patients (>70 years), physical activity was decreased in every time-interval of the year 2020. In time-intervals of eased restrictions, physical activity of younger patients (≤70 years) was not different compared to 2019. No variation in mean heart rate, arrhythmia-burden and count of ICD-therapies was found.

**Conclusion:**

Physical activity shows fluctuations dependent on days of the week and time of the year. During the pandemic, physical activity was reduced in patients with cardiac implantable electronic devices with the strongest reductions during lockdown-periods. Younger patients resumed former levels of physical activity in times of eased restrictions while older patients remained less active. Thus, activation of the elderly population is important to prevent long-term health impairments due to the pandemic.

## 1. Introduction

Severe acute respiratory syndrome coronavirus 2 (SARS-CoV-2) was identified in Wuhan, China in December 2019 [1] with the first case of SARS-CoV-2 in Germany confirmed on January 27^th^ in 2020 [2]. To contain coronavirus disease 2019 (COVID-19) caused by SARS-CoV-2, severe restrictions were imposed prohibiting large parts of occupational, educational and social activities and affecting day-to-day lives of millions of citizens. During COVID-19 pandemic, patients with cardiovascular disease (CD) constitute a subgroup particularly at risk: In case of COVID-19 infection, CD is an independent predictor of mortality risk and progression to severe COVID-19 [3, 4]. Furthermore, physical inactivity, as it may be temporarily enforced by public health interventions, constitutes an independent risk factor for CD itself [5, 6]. The impact of this abrupt and ongoing change of daily life on physical activity of patients with CD and its potential effects on cardiovascular morbidity is not yet conclusively clarified. In addition, the effects of these restrictions may be different or even opposite in different cultural settings, different age groups and different seasonal periods. For that purpose, data from remote monitoring platforms of patients carrying cardiac implantable electronic devices (CIED) can be used. Remote monitoring is part of daily clinical practice and offers the unique possibility to continuously and individually monitor physical activity as well as further parameters such as arrhythmia burden or heart rate (HR) [7]. The objective of this retrospective study is to analyse and quantify the effects of social and occupational restrictions on physical activity in a German cohort of patients suffering from CD during COVID-19 pandemic and to additionally investigate if the number of arrhythmias and implantable cardioverter defibrillator (ICD) therapies is affected.

## 2. Methods

### Study sample

This single centre study included all CIED patients from the University Hospital Würzburg receiving remote monitoring (Home Monitoring, Biotronik SE & Co., KG, Berlin, Germany). CIED encompassed pacemakers (1- and 2-chamber pacemaker, 1-CH-PM, 2-CH-PM, cardiac resynchronisation therapy devices, CRT-P), ICDs (1- and 2-chamber ICD, 1-CH-ICD, 2-CH-ICD), cardiac resynchronisation therapy devices with defibrillator function (CRT-D) and implantable loop recorders (ILR). Device implantation after 01/01/2019 or incomplete data-transfer, defined as missing data transfer in any of the prespecified 12 time intervals within the period analysed (01/01/2019–12/31/2020) led to exclusion from analysis. The study was performed in line with the principles of the Declaration of Helsinki and its later amendments and approval was granted by the Ethics Committee of the University Hospital Würzburg (# 2021-07-1501).

### Data source

General information (age, gender, date of device implantation), indication for device implantation and the type of CIED implanted were taken from the hospital’s documentation system (SAP). Device programming, device derived mean daily physical activity, daily mean heart rate (HR), mean resting HR (rHR), occurrence of arrhythmias (atrial arrhythmia burden, premature ventricular complexes, PVC) and ICD-therapies (anti-tachycardia pacing (ATP), shock-therapy) as well as thoracic impedance (TI) were retrospectively assessed via the remote monitoring platform (Home Monitoring, Biotronik SE & Co., KG, Berlin, Germany). The anonymised dataset is available online (https://github.com/AlexGa/Impact-of-COVID-19-pandemic-on-physical-activity-of-CIED-patients).

### Data analysis

Daily, all CIED monitored remotely transmit a comprehensive data set including technical measurements verifying technical device integrity and clinical data. Episode classification is performed by the arrhythmia detection algorithm of each CIED, though arrhythmia detection thresholds depend on the treating cardiologist’s discretion. Based on alert programming, all CIED transmit automatically if alert thresholds are met, and the patient’s remote monitor is connected. Upon receipt, all alerts are adjudicated by a cardiac device specialist certified by the German Cardiac Society, and further actions are taken depending on clinical need. Patients not transmitting according to their schedule are routinely followed-up via telephone to ensure compliance. Selection of data taken from the remote monitoring system was focused on multimodal assessment of variations of physical activity (physical activity, mean HR, mean rHR) and potentially associated variations of arrhythmia burden (atrial arrhythmia burden, PVC, VT, ventricular fibrillation (VF)). As detection and treatment of VT and VF depend on thresholds programmed at the discretion of the treating cardiologist, only VTs/VF triggering ICD therapies such as ATP or ICD-shocks were included in the analysis to ensure comparability. To investigate age-dependent variations and due to the non-uniform distribution of age, the study population was divided into two subgroups: age group 1 (age ≤ 70 years, n = 77) and age group 2 (age > 70 years, n = 70).

### Time intervals

The period analysed comprised 01/01/2019 until 12/31/2020 to cover two full calendar years. According to important decisions and events related to the COVID-19 pandemic, we defined six time intervals (A20-F20). Prominent news such as the first confirmed case of SARS-CoV-2 in Germany as well as public health interventions of exceptional political and social significance like the imposition of curfews were considered. Time intervals were set as follows: A20: 01/01/20–03/20/20 (absence of public health interventions), B20: 03/21/20–05/06/20 (imposition of curfews, first lockdown), C20: 05/07/2020–08/04/20 and D20: 08/05/20–11/03/20 (stepwise withdrawal of social and occupational restrictions), E20: 11/04/20–12/16/20 (stepwise resumption of public health interventions) and F20: 12/17/20–12/31/20 (reimposition of curfews, second lockdown). To comprise seasonal variations and to allow valid cross comparisons to a regular calendar year, time intervals A20-F20 were identically assigned to the year 2019 (A19-F19).

### Study endpoints

The primary outcomes of the study were physical activity in CIED patients during the 2020 COVID-19 pandemic and the impact of the pandemic on physical activity determined by cross-comparison with corresponding control-periods in 2019. We hypothesized a decline of physical activity in the year of the pandemic. The secondary outcomes were ventricular arrhythmia burden and ICD-therapies in the ICD-subgroup of the study population. An increase of ventricular arrhythmia burden during the 2020 COVID-19 pandemic was expected due to impaired outpatient cardiology care and elevated psychological distress.

### Statistical analysis

The values of each parameter (e.g., physical activity) for each patient in each time interval were calculated as the arithmetic mean over all days per respective time interval. Since we focussed on patients’ physical activities, we only included patients’ data providing at least one day of activity in each time interval. Thus, the basic data set contains information from 147 patients. For the analysis of further parameters, we additionally removed subjects if they did not contain at least one value in each time interval for that particular parameter. To analyse the physical activity within an average week of each time interval, we calculated for each patient the arithmetic means of physical activity at each weekday for all time intervals. We refer to this measure as the mean daily activity (MDA). Calculating the MDA, we considered only subjects who transmitted activity values on each weekday in each time interval. Detecting statistically significant differences between the different time intervals, a paired nonparametric longitudinal data analysis was performed with the R package nparLD [8]. Since the obtained parameters were not normally distributed, we used a nonparametric ANOVA-type-statistic (ATS) [8, 9] based on ranks with repeated measures and a factorial design to model the effects of patient sex, age and time. Due to the non-uniform distribution of age, the two age groups 1 (≤ 70 years) and 2 (> 70 years) were defined. Based on the ATS, pairwise comparisons between time intervals within the years 2019 and 2020 as well as between corresponding time intervals in 2019 and 2020 were performed. Additionally, the ATS was used to quantify statistical differences within and between the years 2019 and 2020. The resulting P-values of all comparisons were corrected for multiple testing with the Benjamini-Yekutieli method. Comparisons showing a corrected P-value below 0.05 were considered statistically significant. The anonymised dataset as well as all codes of the statistical analyses are available online (https://github.com/AlexGa/Impact-of-COVID-19-pandemic-on-physical-activity-of-CIED-patients).

## 3. Results

### Baseline characteristics

248 patients with active home monitoring (Biotronik SE & Co. KG, Berlin, Germany) were considered eligible for inclusion in the current study. 101 patients were excluded from the study population due to device implantation after 01/01/2019, missing data-transfer in any of the 12 time intervals or missing demographic data. The final study cohort consisted of 147 patients. Mean recording of remote monitoring was 660 days. Median age was 69 years ± 11.9 years (Median absolute deviation) and most patients were male (76.2%, n = 112). 92.5% (n = 136) of patients carried an ICD (1-CH-ICD, 2-CH-ICD, CRT-D), 5.4% (n = 8) a PM (2-CH-PM, CRT-P) and 2.0% (n = 3) an ILR. Within the ICD subgroup, indication for ICD implantation was primary prevention in 58.1% (n = 79) and secondary prevention in 41.9% (n = 57). Further demographic and clinical data are displayed in Table 1.

**Table 1:** Baseline characteristics.

	All patients
	n = 147
Age (years)	69 ± 11.9
Male sex	112 (76.2)
**Mean physical activity, % of day**	
2019	12.43 (6.5)
2020	11.49 (6.3)
**Loop recorder**	3 (2.0)
**Pacemaker**	
2CH-PM	3 (2.0)
CRT-P	5 (3.4)
**Defibrillator**	
1CH-ICD	69 (46.9)
2CH-ICD	14 (9.5)
CRT-D	53 (36.1)
**Indication for defibrillator** (n = 136)	
Primary	79 (58.1)
Secondary	57 (41.9)

Age is displayed as median age (median absolute deviation) and further data are absolute numbers (% of patients). CH = chamber, PM = pacemaker, ICD = implantable cardioverter defibrillator, CRT-P = cardiac resynchronisation device w/o defibrillator function, CRT-D = cardiac resynchronisation with defibrillator function.

### Physical activity

#### Physical activity of all CIED patients

**Fig 1:**
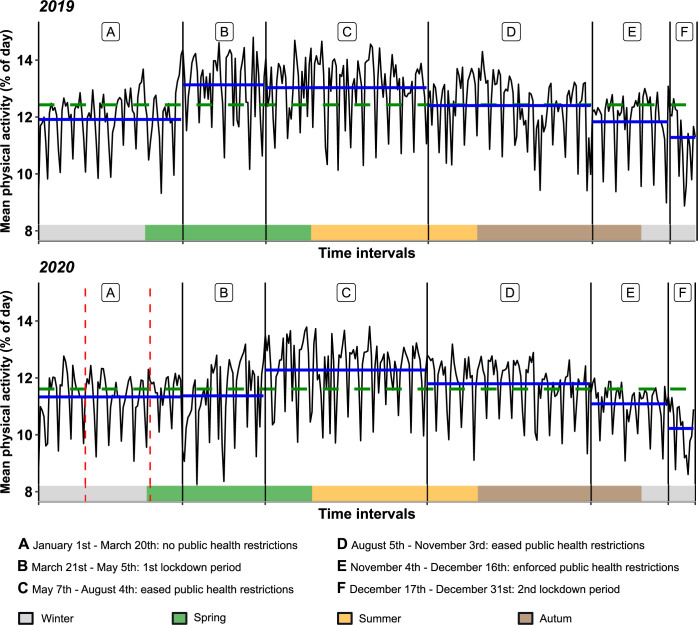
Longitudinal analysis of device derived physical activity in the years 2019 and 2020. Intervals A to F, graphically separated by vertical black lines, indicate time intervals characterized by different levels of public health restrictions in the year 2020 that were then applied analogously to the year 2019. The timepoints of the first case of SARS-CoV-2 confirmed in Germany and of the first case of SARS-CoV-2 confirmed in the city of Würzburg are annotated by vertical red lines. Arithmetic means of device derived physical activity of the years 2019 resp. 2020 are plotted as green dotted horizontal lines. Median of patients’ physical activity within each time interval A-F are displayed as blue horizontal lines. Time periods of the different seasons are displayed graphically as coloured squares (winter: grey, spring: green, summer: yellow, autumn: brown).

**Fig 2:**
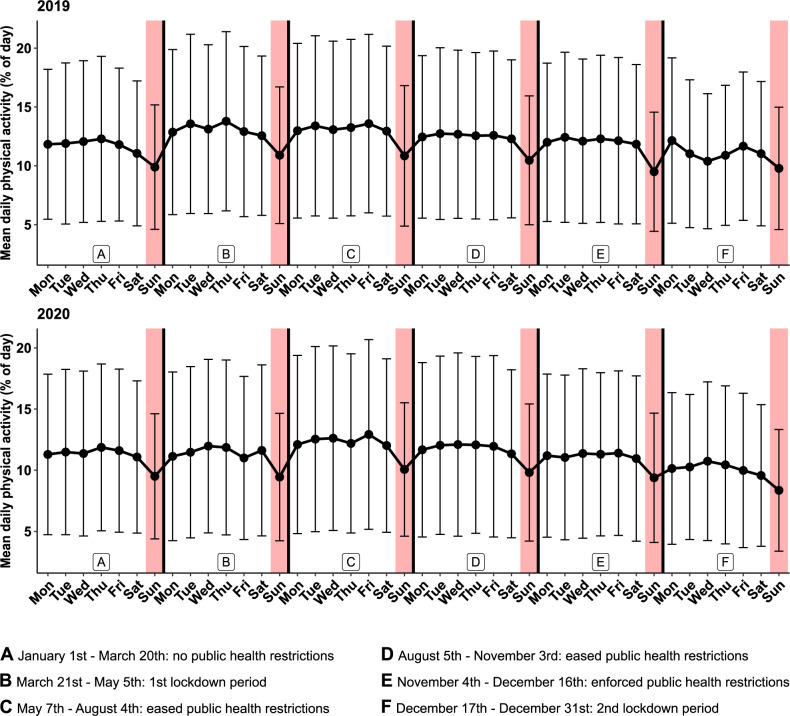
Display of mean daily physical activity (MDA) for each weekday in time intervals A to F in 2019 and 2020. Black dots reflect the arithmetic mean of MDA, calculated over all patients per respective weekday in one time interval. Intervals A to F in the years 2019 and 2020 are displayed. Whiskers show standard deviation (SD) of MDA.

Longitudinal analysis of physical activity of the reference year 2019 shows statistically significant variations. Increase and reduction of physical activity levels in this German study population correlate with changing seasons: As displayed in Fig 1, physical activity is low in winter months, rises in early spring, peaks in early summer, and decreases constantly from late summer to autumn and winter. Interestingly, throughout the entire study period and equally also during the pandemic and lockdown periods, a weekly recurring statistically significant reduction of physical activity on Sundays was observed (Fig 2).

Importantly, mean device derived physical activity was significantly lower in 2020 compared to 2019 (physical activity 2020: 11.49 ± 6.3 vs. 2019: 12.43 ± 6.46; p<0.0001). No gender-dependent variation of mean device derived physical activity was observed neither in 2019 nor in 2020 (physical activity 2019: male vs. female: 12.51 ± 6.59/12.17 ± 6.11; 2020 male vs. female 11.5 ± 6.42/11.47 ± 5.89).

**Fig 3:**
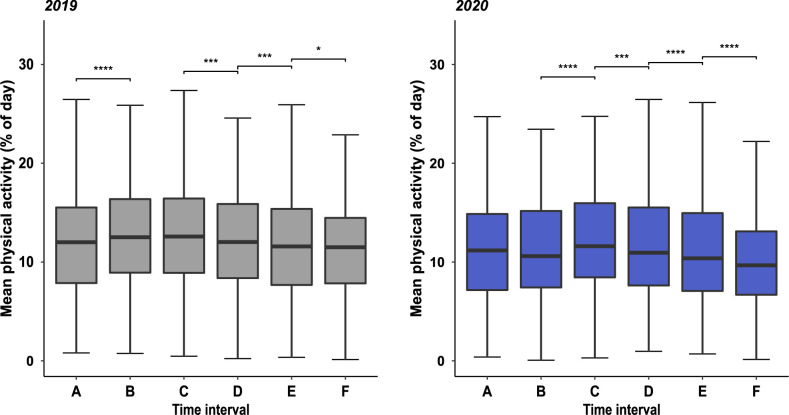
Comparison of physical activity of adjacent time intervals in 2019 and 2020. Box-Whisker-Plots of mean physical activity. Intervals A to F, marking time intervals with different levels of health restrictions in 2020 are annotated and transferred identically to the year 2019. Significant changes in physical activity between adjacent intervals throughout the year are annotated (* = p<0.05, ** = p<0.01, *** = p<0.001, **** = p<0.0001).

**Fig 4:**
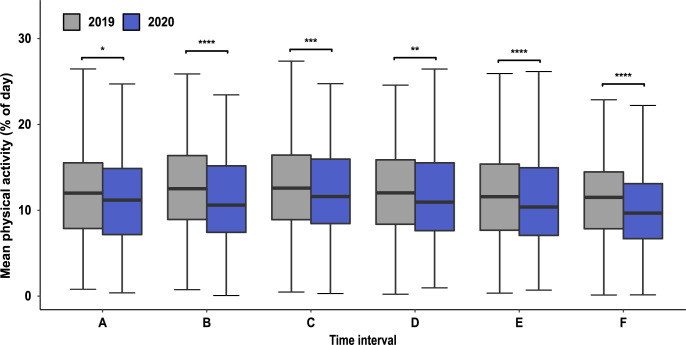
Comparison of physical activity of corresponding time intervals in 2019 and 2020. Box-Whisker-Plot of mean physical activity. Intervals A to F, marking time intervals with different levels of health restrictions in 2020 are annotated and transferred identically to the year 2019. Significant changes in physical activity between corresponding intervals are annotated (* = p<0.05, ** = p<0.01, *** = p<0.001, **** = p<0.0001).

As displayed in Fig 3 and presented in Tables 2 and 3, a significant increase in physical activity was observed in spring 2019 comparing time intervals A and B (physical activity 2019 A vs. B: 12.02 vs. 13.09, fold change (FC) 1.088 (= 8.8%); p < 0.0001), whereas no significant variation in physical activity was seen at the same time period in 2020 when the beginning of spring season was accompanied by the imposition of the first lockdown (physical activity 2020 A vs. B: 11.6 vs.11.59, FC 0.999). As an indicator of a temporal shift, a significant increase in physical activity was observed in 2020 comparing time intervals B and C (physical activity 2020 B vs. C: 11.59/12.32, FC 1.063 (= 6.3%); p < 0.0001) whereas no variation in physical activity was observed in the corresponding time intervals in 2019 (physical activity 2019 B vs. C: 13.09/13.12, FC 1.002). As shown in Fig 3, a constant decrease of physical activity between adjacent time intervals from the climax in early summer in interval C to the nadir of physical activity in winter in interval F was observed in both years. However, in line with the stepwise resumption of public health interventions in interval E and reimposition of the second lock-down in interval F, inter-interval changes of physical activity between adjacent intervals (C–F) were more pronounced in 2020 (Tables 2 and 3). As presented in Fig 4, comparing corresponding time intervals of 2019 and 2020, physical activity was lower in every interval of the year 2020 with the greatest differences in intervals B: FC 0.885 (= 11.5%), p<0.0001, E: FC 0.924 (= 7.6%), p<0.0001 and F: FC 0.889 (= 11.1%), p<0.0001, reflecting the two lockdown periods (intervals B and F) and the time interval of stepwise resumption of public health restrictions (interval E).

**Table 2:** Comparison of physical activity of adjacent time intervals in 2019.

Time interval	PA	FC / rcPA	p-value
		
A19	12.02 ± 6.17	1.088 / 8.8	<0.0001
B19	13.09 ± 6.62		
B19	13.09 ± 6.62	1.002 / 0.2	n.s.
C19	13.12 ± 6.77		
C19	13.12 ± 6.77	0.958 / -4.2	<0.01
D19	12.57 ± 6.54		
D19	12.57 ± 6.54	0.967 / -3.3	<0.001
E19	12.15 ± 6.56		
E19	12.15 ± 6.56	0.957 / -4.3	<0.05
F19	11.63 ± 6.17		

P-values refer to differences of mean physical activity (PA) of adjacent time intervals (A-F). Fold changes (FC) i.e., relative changes of physical activity (rcPA) for each pair of adjacent time intervals are displayed.

**Table 3:** Comparison of physical activity of adjacent time intervals within 2020.

Time interval	PA	FC / rcPA	p-value
		
A20	11.60 ± 6.29	0.999 / 0.1	n.s.
B20	11.59 ± 6.38		
B20	11.59 ± 6.38	1.063 / 6.3	<0.0001
C20	12.32 ± 6.48		
C20	12.32 ± 6.48	0.965 / -3.5	<0.001
D20	11.89 ± 6.37		
D20	11.89 ± 6.37	0.944 / -5.6	<0.0001
E20	11.23 ± 6.34		
E20	11.23 ± 6.34	0.921 / -7.9	<0.0001
F20	10.34 ± 5.81		

P-values refer to differences of mean physical activity (PA) of adjacent time intervals (A-F). Fold changes (FC) i.e., relative changes of physical activity (rcPA) for each pair of adjacent time intervals are displayed.

#### Physical activity of CIED patients by age groups

Physical activity and patients’ response to changing social and occupational conditions may be age dependent. Due to the non-uniform distribution of age, the study cohort was subdivided in age group 1 (≤ 70 years) and age group 2 (> 70 years). Comparing age group 1 and 2, mean physical activity (physical activity 2019: age group 1 vs. age group 2: 14.51 ± 7.12 vs. 10.15 ± 4.75, p < 0.0001; physical activity 2020: age group 1 vs. age group 2: 13.65 ± 6.81 vs. 9.13 ± 4.65, p < 0.0001) was significantly higher in the younger subgroup in 2019 as well as in 2020.

**Fig 5:**
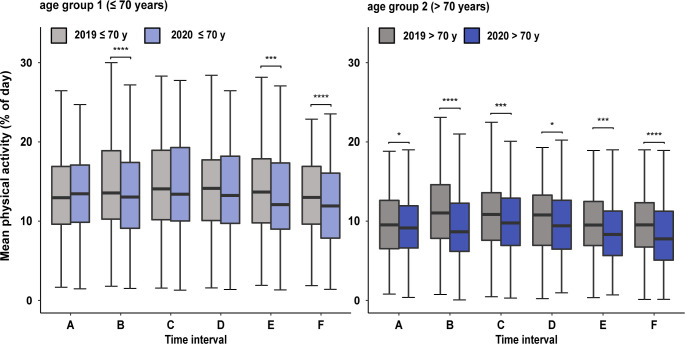
Comparison of physical activity of corresponding time intervals in 2019 and 2020 by age groups. Box-Whisker plots of mean physical activity. Age group 1 < 70 years, age group 2 ≥ 70 years. Intervals A to F, marking time intervals with different levels of health restrictions in 2020 are annotated and transferred identically to the year 2019. Significant changes in physical activity between corresponding intervals of 2019 and 2020 are annotated (* = p<0.05, ** = p<0.01, *** = p<0.001, **** = p<0.0001).

Comparing the corresponding time intervals of 2019 and 2020 after subdivision into age categories, physical activity was significantly decreased in every time interval of the year 2020 in older patients (age group 2) as displayed in Fig 5 and presented in Table 4. In contrast, physical activity of younger patients (age group 1) was only significantly decreased in time intervals marked by strict public health interventions (intervals B20, E20 and F20) and physical activity was similar to 2019 in time intervals marked by missing (interval A20) or eased public health interventions (intervals C20 and D20). (Fig 5, Table 4).

**Table 4:** Comparison of physical activity between corresponding time intervals of years 2019 and 2020 in all patients and in different age groups.

	All patients			Age group 1 (≤ 70 years)			Age group 2 (> 70 years)		
Time interval	PA	FC / rcPA	p-value	PA	FC / rcPA	p-value	PA	FC / rcPA	p-value
A19	12.02 ± 6.17	0.964 / -3.6	<0.05	14.05 ± 6.76	0.986 / -1.4	n.s.	9.8 ± 4.54	0.930 / 7.0	<0.05
A20	11.60 ± 6.29			13.86 ± 6.89			9.11 ± 4.4		
B19	13.09 ± 6.62	0.885 / -11.5	<0.0001	15.01 ± 7.37	0.904 / -9.6	<0.0001	10.98 ± 4.94	0.857 / -14.3	<0.0001
B20	11.59 ± 6.38			13.57 ± 6.88			9.41 ± 4.99		
C19	13.12 ± 6.77	0.939 / -6.1	<0.001	15.15 ± 7.45	0.953 / -4.7	n.s.	10.88 ± 5.1	0.917 / -8.3	<0.001
C20	12.32 ± 6.48			14.45 ± 6.92			9.97 ± 5.05		
D19	12.57 ± 6.54	0.946 / -5.4	<0.001	14.69 ± 7.23	0.960 / -4.0	n.s.	10.24 ± 4.72	0.924 / -7.6	<0.05
D20	11.89 ± 6.37			14.10 ± 6.94			9.46 ± 4.63		
E19	12.15 ± 6.56	0.924 / -7.6	<0.0001	14.42 ± 7.18	0.934 / -6.6	<0.001	9.66 ± 4.71	0.908 / -9.2	<0.001
E20	11.23 ± 6.34			13.47 ± 6.94			8.76 ± 4.51		
F19	11.63 ± 6.17	0.889 / -11.1	<0.0001	13.72 ± 6.81	0.907 / -9.3	<0.0001	9.34 ± 4.38	0.862 / -13.8	<0.0001
F20	10.34 ± 5.81			12.43 ± 6.31			8.04 ± 4.16		

P-values refer to differences of mean physical activity (PA) of corresponding time intervals (A-F) in 2019 and 2020. Fold changes (FC) i.e., relative changes of physical activity (rcPA) for each pair of corresponding time intervals are displayed.

### Mean HR, mean rHR, atrial arrhythmia burden and thoracic impedance

Physical exertion leads to an increase in HR in chronotropic competent patients as well as in chronotropic incompetent patients with activated rate-adaptive pacing-modes. Furthermore, rHR is affected by training condition. Therefore, we additionally analysed mean HR and mean rHR in the respective time intervals of both years. Contrary to our assumption, no significant variation of mean HR and mean rHR was found comparing 2019 and 2020 (mean HR: 2019 69.5 bpm, 2020 68.8; mean rHR: 2019 58.6 bpm, 2020 58.2 bpm). Comparing corresponding time intervals of 2019 and 2020, a tendency towards numerically lower mean HR and mean rHR in time intervals A20 –F20 was observed. However, even after subdivision into age categories, no significant differences between intervals of mean HR and mean rHR was detected. Additional evaluation of atrial arrhythmia burden and thoracic impedance revealed no significant differences across years and age groups.

### Ventricular arrhythmias and ICD therapies

As variation of physical activity levels may translate into changes of electric stability in ICD carriers, we investigated the frequency of ICD therapies in the respective time intervals. Within the ICD subgroup a cumulative number of 99 ICD therapies were observed in 26 patients in 2019 (nATP = 75, nICD-shock = 24). In 2020, a cumulative number of 36 ICD therapies (nATP = 24, nICD-shock = 12) were recorded in 25 patients. Despite numerically differences, no significant variation in ICD therapies (1: ATP, 2: ICD shocks, 3: ATP and ICD shocks combined) comparing time intervals in 2020 and 2019 was detected.

## 4. Discussion

This is a retrospective, observational cohort study assessing physical activity and ventricular arrhythmia burden in CIED patients via remote monitoring over a nearly two-year period in Germany. The following key observations could be made: 1) In this cohort of 147 CIED-patients, changes of physical activity showed a distinct weekly periodicity and seasonal pattern. 2) During COVID-19 pandemic, physical activity was significantly reduced with the most pronounced reduction during the two lockdown periods. 3) Younger CIED patients resumed their former level of physical activity in times of eased public health restrictions whereas 4) older patients remained less active even after lockdown periods. 5) Despite numeric reductions, no statistically significant decrease in ICD therapies was observed during COVID-19 pandemic.

### Fluctuations in physical activity on different days of the week and throughout the year

Longitudinal analysis of physical activity revealed a weekly recurring reduction of physical activity on Sundays. This finding indicates that patients in this CIED-cohort do not use leisure time for physically strenuous activities. Furthermore, levels of physical activity change throughout the year in a typical pattern that correlates with different seasons: Physical activity levels peak in early spring and are lowest in winter months. Thus, regardless of a pandemic, patients are at greater risk of losing their physical condition in the winter months.

### Impact of public health restrictions on physical activity in different countries and population cohorts

We show a relative reduction of physical activity of 11.5% during the first lockdown period in comparison to the 2019 control-interval and constant levels of physical activity in comparison to the 2020 pre-lockdown time interval. Thus, the usual increase in physical activity in spring was blunted in 2020. Comparing corresponding time intervals of 2019 and 2020, physical activity was decreased in every time interval of the year 2020 with the greatest differences in physical activity in time intervals of strictest public health interventions. These results stand in line with two observational remote-monitoring trials from Italy. Whereas the first study detected a relative reduction of physical activity of 21.6% in 180 ICD/CRT patients during two consecutive intervals pre and post imposition of curfews [10], the second study even found a relative reduction of physical activity of 25.9% analysing 211 ICD/CRT patients during a 16-week lockdown period [11]. The lower reduction of physical activity in our study may be explained by the fact that Italy and its health system were affected even more severely during the first wave of the pandemic, which resulted in earlier and stricter containment measures [12, 13]. Furthermore, climate differences cause a later rise in activity levels during the year in central Europe. Whereas existing studies are limited to the first wave of COVID-19 pandemic [10, 11, 14, 15], our analysis covers the entire year 2020 including time-periods of eased restrictions and a second lockdown period and thus reflects how the pandemic evolved throughout the year. Importantly, in times of eased restrictions, activity levels remained depressed in the study population, implicating a persistent change of daily life attributed to the pandemic. Moreover, our study is the first study that analyses the effect of COVID-19 pandemic on physical activity of CIED patients by cross-comparison to a regular calendar year that comprises seasonal variations.

### Impact of public health restrictions on physical activity in different age groups

Our study shows distinct age-dependent differences in activity levels: During the pandemic, younger CIED patients quickly resumed their former level of physical activity in times of eased restrictions, whereas older patients remained less active even after lockdown periods. These results are interesting as they contradict a previous study performed in Italy that reported a more prominent and more consistent reduction of physical activity in patients aged less than 70 years compared to older patients. We attribute the differences to the fact that governmental restrictions in Italy were stricter with regard to employed people. In line with this, the Stringency index, objectifying the severity of governmental containment measures, consistently showed lower scores for Germany compared to Italy [12]. We attribute the age-specific differences in our study cohort primarily to more pronounced behavioural changes in the older population, that faces a significantly increased risk of morbidity and mortality in the event of SARS-CoV-2 infection [16]. Furthermore, it must be noted that the time period analysed by the study performed in Italy was comparatively short, comprised solely one lockdown period and did not comprise seasonal variations [10].

### Impact of physical activity on ventricular arrhythmias and ICD-therapies

The seemingly paradoxical detrimental and/or beneficial effects of exercise training on ICD-therapies are well described: While physical exertion can acutely trigger arrhythmias during a hyperadrenergic state, it is known to reduce mortality and ICD-therapies in the long-term [17–19]. Therefore, it is well conceivable, that an abrupt decrease of physical activity may acutely reduce ventricular arrhythmia burden in the short-term. In accordance with these findings, we observed a numeric reduction in cumulative ICD-therapies that was related to the total decrease in device-detected physical activity. This finding is corroborated by previous data showing reduced ventricular arrhythmia burden in ICD-patients during the pandemic: A recent observational cohort study assessing ventricular arrhythmias in almost 6000 ICD-patients found a 32% reduction in ventricular arrhythmias during the COVID-19 pandemic [20]. Unfortunately, no information about the levels of physical activity in the context of reduced ventricular arrhythmia burden was provided. Hinting towards a potential association of reduced levels of physical activity and reduced arrhythmia burden during the pandemic, a single-centre retrospective study that analysed physical activity in ICD/CRT-patients described a trend towards reduced numbers of VTs, VF and occurrence of ATP along with a significant reduction of physical activity [11]. However, the relevance of this observation is limited due to a highly skewed data distribution.

Whether reduced levels of physical activity and a reduced number of ICD therapies are coincidental or causally related cannot be dissolved yet. It is important, however, that exercise training has proven to reduce ICD shocks in the long term [18]. Likewise, the beneficial effects of exercise training on cardiovascular health and cardiovascular risk constitute a cornerstone of primary and secondary health prevention [21]. Therefore, we are convinced that the negative impacts of physical inactivity will outweigh potential positive short-term effects and will increase cardiovascular risk and ventricular arrhythmia burden in the long run.

### Implications of this study

Our study shows that CIED patients in general are at increased risk of losing their physical fitness level during the pandemic. Given the persistent and strongest reduction of physical activity levels among the elderly, motivation and guided activation of this vulnerable population are paramount to prevent further health impairments during the ongoing pandemic. Treating physicians should encourage patients to stay active and help them find ways to perform physical activities that are compatible with social restrictions and curfews. Furthermore, future studies are necessary to investigate long-term effects of physical inactivity during the pandemic on cardiovascular health in general and arrhythmia risk in particular.

## 5. Limitations

Our results should be considered in light of two limitations. First, the study comprises a heterogeneous population of patients as carriers of different types of CIEDs (ICD, CRT-D, PM, ILR) were enrolled. A differentiated analysis of the different subgroups (defibrillator vs. PM vs. ILR) was not feasible due to the small number of patients in the PM- and ILR-subgroup. Thus, we cannot exclude an impact of the type of CIED on the study’s results. Second, in this remote-monitoring study, important baseline characteristic such as ejection fraction and NYHA functional class were not reported. Therefore, an impact of these variables on the study’s results cannot be excluded.
